# Revisiting the Central Metabolism of the Bloodstream Forms of *Trypanosoma brucei*: Production of Acetate in the Mitochondrion Is Essential for Parasite Viability

**DOI:** 10.1371/journal.pntd.0002587

**Published:** 2013-12-19

**Authors:** Muriel Mazet, Pauline Morand, Marc Biran, Guillaume Bouyssou, Pierrette Courtois, Sylvie Daulouède, Yoann Millerioux, Jean-Michel Franconi, Philippe Vincendeau, Patrick Moreau, Frédéric Bringaud

**Affiliations:** 1 Centre de Résonance Magnétique des Systèmes Biologiques (RMSB), UMR5536, Université Bordeaux Segalen, CNRS, Bordeaux, France; 2 Laboratoire de Biogenèse Membranaire, UMR5200 Université Bordeaux Segalen, CNRS, Bâtiment A3, INRA Bordeaux Aquitaine, Villenave d'Ornon, France; 3 Laboratoire de Parasitologie, UMR177 IRD CIRAD, Université Bordeaux Segalen, BP 43, Bordeaux, France; McGill University, Canada

## Abstract

**Background:**

The bloodstream forms of *Trypanosoma brucei*, the causative agent of sleeping sickness, rely solely on glycolysis for ATP production. It is generally accepted that pyruvate is the major end-product excreted from glucose metabolism by the proliferative long-slender bloodstream forms of the parasite, with virtually no production of succinate and acetate, the main end-products excreted from glycolysis by all the other trypanosomatid adaptative forms, including the procyclic insect form of *T. brucei*.

**Methodology/Principal Findings:**

A comparative NMR analysis showed that the bloodstream long-slender and procyclic trypanosomes excreted equivalent amounts of acetate and succinate from glucose metabolism. Key enzymes of acetate production from glucose-derived pyruvate and threonine are expressed in the mitochondrion of the long-slender forms, which produces 1.4-times more acetate from glucose than from threonine in the presence of an equal amount of both carbon sources. By using a combination of reverse genetics and NMR analyses, we showed that mitochondrial production of acetate is essential for the long-slender forms, since blocking of acetate biosynthesis from both carbon sources induces cell death. This was confirmed in the absence of threonine by the lethal phenotype of RNAi-mediated depletion of the pyruvate dehydrogenase, which is involved in glucose-derived acetate production. In addition, we showed that *de novo* fatty acid biosynthesis from acetate is essential for this parasite, as demonstrated by a lethal phenotype and metabolic analyses of RNAi-mediated depletion of acetyl-CoA synthetase, catalyzing the first cytosolic step of this pathway.

**Conclusions/Significance:**

Acetate produced in the mitochondrion from glucose and threonine is synthetically essential for the long-slender mammalian forms of *T. brucei* to feed the essential fatty acid biosynthesis through the “acetate shuttle” that was recently described in the procyclic insect form of the parasite. Consequently, key enzymatic steps of this pathway, particularly acetyl-CoA synthetase, constitute new attractive drug targets against trypanosomiasis.

## Author Summary

Many protists, including parasitic helminthes, trichomonads and trypanosomatids, produce acetate in their mitochondrion or mitochondrion-like organelle, which is excreted as a main metabolic end-product of their energy metabolism. We have recently demonstrated that mitochondrial production of acetate is essential for fatty acid biosynthesis and ATP production in the procyclic insect form of *T. brucei*. However, acetate metabolism has not been investigated in the long-slender bloodstream forms of the parasite, the proliferative forms responsible for the sleeping sickness. In contrast to the current view, we showed that the bloodstream forms produce almost as much acetate from glucose than the procyclic parasites. Acetate production from glucose and threonine is synthetically essential for growth and *de novo* synthesis of fatty acids of the bloodstream trypanosomes. These data highlight that the central metabolism of the bloodstream forms contains unexpected essential pathways, although minor in terms of metabolic flux, which could be targeted for the development of trypanocidal drugs.

## Introduction

*Trypanosoma brucei* is a unicellular eukaryote, belonging to the protozoan order Kinetoplastida that causes sleeping sickness in humans and economically important livestock diseases [1]. This parasite undergoes a complex life cycle during transmission from the bloodstream of a mammalian host (bloodstream forms of the parasite - BSF) to the alimentary tract (procyclic form - PF) and salivary glands (epimastigote and metacyclic forms) of a blood feeding insect vector, the tsetse fly. In the bloodstream of the mammalian host, the pleomorphic BSF strains proliferate as “long-slender” BSF (LS-BSF) and differentiate into the non-proliferative “short-stumpy” trypanosomes (SS-BSF), which are preadapted for differentiation into PF in the insect midgut [2]. The environmental changes encountered by the parasite require significant morphological and metabolic adaptations, as exemplified by important qualitative and quantitative differences in glucose metabolism between BSF and PF [3], [4].

**Figure 1:**
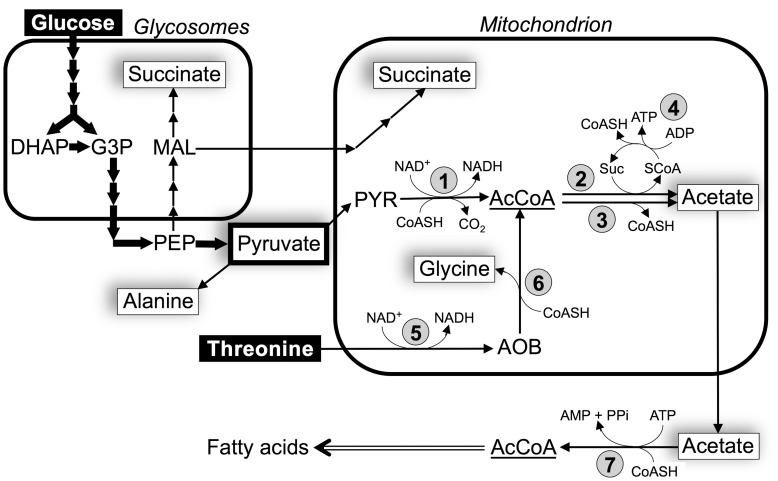
Schematic representation of acetate production from glucose and threonine in BSF. Black arrows indicate enzymatic steps of glucose and threonine metabolism and the double line arrow represents *de novo* biosynthesis of fatty acids. Excreted end-products of metabolism of glucose and threonine are boxed. The thick arrows illustrate the high glycolytic flux leading to production of pyruvate, which accounts for 85.1% of the excreted end-products from glucose metabolism. Abbreviations: AcCoA, acetyl-CoA; AOB, amino oxobutyrate; DHAP, dihydroxyacetone phosphate; G3P, glyceraldehyde 3-phosphate; MAL, malate; PEP, phosphoenolpyruvate; PYR, pyruvate; SCoA, succinyl-CoA. Indicated enzymes are: 1, pyruvate dehydrogenase complex (PDH); 2, acetate∶succinate CoA-transferase (ASCT); 3, acetyl-CoA thioesterase (ACH); 4, succinyl-CoA synthetase (SCoAS); 5, threonine 3-dehydrogenase (TDH); 6, 2-amino-3-ketobutyrate coenzyme A ligase (AKCT); 7, acetyl-CoA synthetase (AMP-dependent enzyme, AceCS).

PF living in the tsetse fly midgut – where glucose is scarce or absent – have developed an elaborate energy metabolism based on amino acids, such as proline. However, when grown in standard glucose-rich conditions, they prefer glucose to proline as a carbon source [5], [6]. PF converts glucose into the partially oxidized and excreted end-products, acetate and succinate, with most of the glycolysis taking place in specialized peroxisomes called glycosomes [7]. In the course of glycolysis, phosphoenolpyruvate (PEP) is produced in the cytosol, where it is located at a branching point to feed the glycosomal ‘succinate branch’ and the mitochondrial ‘acetate and succinate branches’ (see Fig. 1). For the “succinate branches”, PEP must re-enter the glycosomes where it is converted into malate and succinate within that compartment. Malate, which moves from the glycosomes into the mitochondrion, can also be converted into succinate therein. Additionally, PEP can be converted in the cytosol into pyruvate to feed the ‘acetate branch’ (steps 1–4 in Fig. 1). In the mitochondrion, pyruvate is converted by the pyruvate dehydrogenase complex (PDH, EC 1.2.4.1, step 1) into acetyl-CoA and then into acetate by two different enzymes, *i.e.* acetate∶succinate CoA transferase (ASCT, EC 2.8.3.8, step 2) and acetyl-CoA thioesterase (ACH, EC 3.1.2.1, step 3) [8]–[10]. In PF, acetate production plays an important role for mitochondrial ATP production by the ASCT/SCoAS cycle (steps 2 and 4), while ACH is not involved in ATP production [10]. Acetate can also be produced from threonine, a major carbon source of PF present in the *in vitro* medium [6], [11], [12]. This amino acid is converted into acetate by threonine-3-dehydrogenase (TDH, EC 1.1.1.103, step 5), acetyl-CoA∶glycine C acetyltransferase (EC 2.3.1.29, step 6) and probably ASCT and/or ACH. We recently showed that PF uses a new metabolic pathway only observed in PF trypanosomes so far, named the “acetate shuttle”, which transfers acetyl-CoA from the mitochondrion to the cytosol to feed the essential cytosolic fatty acid biosynthesis [13]. In this shuttle, acetate produced in the mitochondrion from acetyl-CoA is exported in the cytosol and converted back into acetyl-CoA by the cytosolic acetyl-CoA synthetase (AMP-dependent enzyme, AceCS, EC 6.2.1.1, step 7).

In contrast to PF, BSF trypanosomes rely only on glucose for their energy production, with a 5- to 10-fold higher rate of glucose consumption [14]. It is generally accepted that the proliferative LS-BSF grown under aerobiosis convert glucose exclusively into pyruvate [15], [16], although excretion of trace amounts of other incompletely oxidized end-products such as glycerol, succinate and alanine have been reported [14], [17]. These minor glycolytic end-products are thought to be produced by “contaminating” non-proliferative SS-BSF trypanosomes that have developed a more elaborated central metabolism with a number of PF traits, including production of acetate and succinate from glycolysis [18], [19]. Consequently, recent reports consider that LS-BSF trypanosomes do not produce acetate from glucose. In the seventies, acetate production was reported from the threonine degradation in both PF and BSF trypanosomes [12], however, this metabolic pathway was not further investigated.

Here we investigated the role of glucose and threonine degradation in acetate production in the monomorphic 427 BSF strain, which proliferates as LS-BSF trypanosomes and has lost the ability to differentiate into non-proliferative SS-BSF [20], [21]. This BSF cell line produces and excretes acetate, as a minor end-product of glucose metabolism, with a metabolic flux in the same range as observed for PF. Glucose and threonine contribute almost equally to acetate production, which is essential for the viability of proliferative BSF trypanosomes, as demonstrated by reverse genetics approaches. Our data reveal unexpected metabolic similarities between PF and LS-BSF trypanosomes.

## Materials and Methods

### Growth and maintenance of trypanosomes

The bloodstream form of T. brucei 427 90-13 (TetR-HYG T7RNAPOL-NEO), a 427 221a line (MiTat 1.2) designed for the conditional expression of genes, was cultured at 37°C in IMDM (*Iscove's Modified Dulbecco's Medium, Life Technologies*) supplemented with 10% (v/v) heat-inactivated fetal calf serum (FCS), 0.25 mM ß-mercaptoethanol, 36 mM NaHCO_3_, 1 mM hypoxanthine, 0.16 mM thymidine, 1 mM sodium pyruvate, 0.05 mM bathocuprone and 2 mM L-cysteine [22]. To prepare threonine-depleted IMDM medium all the compounds constituting the medium, except threonine, were purchased from Sigma-Aldrich. The procyclic form of T. brucei EATRO1125 was cultured at 27°C in SDM79 medium containing 10% (v/v) heat-inactivated fetal calf serum and 35 µg/mL hemin [23].

### Gene knock out of TDH

Replacement of the threonine-3-dehydrogenase (*TDH*: Tb927.6.2790) by the puromycin (*PAC*) and blasticidin (*BSD*) resistance markers *via* homologous recombination was performed with DNA fragments containing a resistance marker gene flanked by the TDH UTR sequences. The TDH knock out was generated in the 427 90-13 BSF parental cell line, which constitutively expresses the T7 RNA polymerase gene and the tetracycline repressor under the control of a T7 RNA polymerase promoter for tetracycline inducible expression (*TetR-HYG T7RNAPOL-NEO*) [24]. Transfection and selection of drug-resistant clones were performed as previously reported using the Nucleofactor system [25]. The first and second *TDH* alleles were replaced by puromycin- and blasticidin-resistant genes, respectively. Transfected cells were selected in IMDM medium containing hygromycin B (5 µg/mL), neomycin (2.5 µg/mL), puromycin (0.1 µg/mL) and blasticidin (10 µg/mL). The selected cell line (*TetR-HYG T7RNAPOL-NEO Δtdh::PAC/Δtdh::BSD*) is called Δ*tdh*.

### Inhibition of gene expression by RNAi

Accession numbers (http://www.genedb.org/genedb/tryp/) of genes targeted by RNAi, acetyl-CoA synthetase (AMP-dependent enzyme, AceCS) and E2 subunit of the pyruvate dehydrogenase complex (PDH-E2), are Tb927.8.2520 and Tb927.10.7570, respectively. RNAi-mediated inhibition of gene expression in the 427 90-13 BSF parental cell line was performed by expression of stem-loop “sense/anti-sense” RNA molecules of the targeted sequences introduced into the pHD1336 (kindly provided by C. Clayton, ZMBH, Heidelberg, Germany, cclayton@zmbh.uni-heidelberg.de). The AceCS-SAS and PDH-E2-SAS “sense/anti-sense” constructs were first generated in the pLew100 vector (kindly provided by E. Wirtz and G. Cross) [24] as described before [5], [13]. Then the AceCS-SAS and PDH-E2-SAS HindIII-BamHI cassettes extracted from the pLew100 plasmids were inserted in HindIII-BamHI digested pHD1336 vector, which contains the blasticidin resistance gene. The RNAi-harboring *^RNAi^*AceCS and *^RNAi^*PDH single mutant cell lines were produced by transfection of the 427 90-13 cell line with the NotI-linearized pHD-AceCS-SAS and pHD-PDH-E2-SAS plasmids, respectively, and selected in IMDM medium containing hygromycin B (5 µg/mL), neomycin (2.5 µg/mL) and blasticidin (10 µg/mL). For transfection of the Δ*tdh* cell line with the pLew-PDH-E2-SAS construct, all of the four antibiotics used to select the Δ*tdh* cell line, in addition to phleomycin (2.5 µg/mL), were included in the medium to select double mutant cell lines.

### Production of TDH antibodies

A recombinant fragment containing the full-length *TDH* gene was inserted into the NdeI and BamHI restriction sites of the pET28a expression vectors (Novagen) to express in BL21 *Escherichia coli* the TDH protein preceded by a N-terminal histidine tag (6 histidine codons). Cells were harvested by centrifugation, and recombinant proteins purified by nickel chelation chromatography (Novagen) from the insoluble fraction according to the manufacturer's instructions. The anti-TDH immune serum was raised in rabbits by five injections at 15-day intervals of 100 µg of TDH-His recombinant nickel-purified proteins, emulsified with complete (first injection) or incomplete Freund's adjuvant (Proteogenix S.A.). Antibodies raised against the *T. brucei* TDH protein expressed in *E. coli* recognize a single 36.5 kDa protein in western blots, corresponding to the calculated TDH molecular weight (36.96 kDa).

### Western blot analyses

Total protein extracts of bloodstream or procyclic forms of *T. brucei* (5×10^6^ cells) were separated by SDS PAGE (10%) and immunoblotted on Immobilon-P filters (Millipore) [26]. Immunodetection was performed as described [26], [27] using as primary antibodies, the mouse anti-sera against AceCS diluted 1∶100 [13], PDH-E2 diluted 1∶500 [28] or the heat shock protein 60 (hsp60) diluted 1∶10,000 [29], or the rabbit anti-sera against TDH diluted 1∶500, acetate∶succinate CoA-transferase (ASCT) diluted 1∶100 [9], acetyl-CoA thioesterase (ACH) diluted 1∶500 [10] or glycerol-3-phosphate dehydrogenase (GPDH, EC 1.1.1.8) diluted 1∶100 [30]. Goat anti-rabbit Ig/peroxidase (1∶10,000 dilution) or goat anti-mouse Ig/peroxidase were used as secondary antibody and revelation was performed using the SuperSignal West Pico Chemiluminescent Substrate as described by the manufacturer (Thermo Scientific). Images were acquired and analyzed with a KODAK Image Station 4,000 MM and quantitative analyses were performed with the KODAK MI application.

### Enzyme assays

Cells were washed in PBS and lysed by sonication (5 sec at 4°C) in hypotonic lysis buffer (5 mM Na_2_HPO_4_, 0.3 mM KH_2_PO_4_). Determination of TDH and PDH enzymatic activities was performed using a spectrophotometric assay as described before [12], [31], [32].

### Immunofluorescence analysis

To stain mitochondria of the wild-type cell lines, 200 nM MitoTracker Red CMXRos (Invitrogen) were added to the culture, followed by a 20 min incubation and washes in PBS. Then wild-type cells were fixed with 4% formaldehyde in PBS, permeabilized with 1% Triton X-100, and spread on poly-L-lysine-coated slides. The slides were then incubated for 45 min in PBS containing 5% BSA, followed by incubation in PBS with 2% BSA and the primary antiserum, 1∶50 diluted rabbit anti-TDH, mouse anti-AceCS or mouse anti-PDH-E1α. After washing with PBS, the slides were incubated with 2 µg/mL Alexa 594 anti-rabbit IgG conjugate or Alexa Fluor 594 anti-mouse IgG conjugate (Molecular Probes). Slides were then washed and mounted in the SlowFade antifade reagent (Invitrogen). Cells were visualized with a Leica DM5500B microscope, and images were captured by an ORCA-R2 camera (Hamamatsu) and Leica MM AF Imaging System software (MetaMorph).

### NMR experiments

The bloodstream forms (2.5×10^7^ cells, ∼0.25 mg of proteins) or procyclic form (5×10^7^ cells, ∼0.25 mg of proteins) of *T. brucei* were collected by centrifugation at 1,400 g for 10 min, washed once/twice with phosphate-buffered saline (PBS) and incubated for 5 h at 37°C in 2.5 mL of incubation buffer (PBS supplemented with 5 g/L NaHCO_3_, pH 7.4), with [U-^13^C]-glucose (4 mM) in the presence or the absence of threonine (4 mM). The same experiments were performed with regular ^12^C glucose as the only carbon source. The integrity of the cells during the incubation was checked by microscopic observation. 50 µL of maleate (20 mM) were added as internal reference to a 500 µL aliquot of the collected supernatant and proton NMR (^1^H-NMR) spectra were performed at 125.77 MHz on a Bruker DPX500 spectrometer equipped with a 5 mm broadband probe head. Measurements were recorded at 25°C with an ERETIC method. This method provides an electronically synthesized reference signal [33]. Acquisition conditions were as follows: 90° flip angle, 5,000 Hz spectral width, 32 K memory size, and 9.3 sec total recycle time. Measurements were performed with 256 scans for a total time close to 40 min. Before each experiment, the phase of the ERETIC peak was precisely adjusted. Resonances of the obtained spectra were integrated and results were expressed relative to ERETIC peak integration. The linear production of pyruvate and acetate throughout the experiment was confirmed by ^1^H-NMR quantification of the end-products excreted by the wild type trypanosomes incubated for 6 h in PBS containing 4 mM [U-^13^C]-glucose (data not shown).

### Fatty acid labeling from [U-^14^C]-acetate

Cells in the late exponential phase (5×10^7^ cells) were incubated for 16 h in 10 mL of modified IMDM medium without threonine, pyruvate, leucine, isoleucine, valine, containing 25 mM glucose, 100 µM acetate and 40 µCi of [1-^14^C]-acetate (55.3 mCi/mmol). Cells were checked microscopically for viability several times during incubation. Subsequently, lipids were extracted by chloroform∶methanol (2∶1, v/v) for 30 min at room temperature, and then washed three times with 0.9% NaCl. The washed lipid extracts were then evaporated and lipids were dissolved in 1 mL of methanol∶H_2_SO_4_ (40∶1, v/v). Trans-esterification of the fatty acids of the lipids was performed at 80°C for 60 min. After cooling the samples, 400 µL of hexane (99% pure) and 1.5 mL of H_2_O were added, and the mixture was homogenized vigorously for 20 sec. The samples were then centrifuged for 5 min at 1,000 g to separate the phases, and the hexane upper phases containing fatty acid methyl ester (FAMEs) were recovered without contact with the lower phases. FAMEs were loaded onto HPTLC plates developed in hexane/ethylether/acetic acid (90∶15∶2, v/v) and were separated (R_F_ 0.90). They were identified by co-migration with known standards. Their radio-labeling was then determined with a STORM 860 (GE Healthcare). The values were normalized with the amounts of total esters in each sample and detected by densitometry analysis using a TLC scanner 3 (CAMAG, Muttenz, Switzerland) as already described [13].

### *In vivo* experiments

Eight- to ten-week-old female BALB/c mice bred at the SAS Centre d'Elevage Depré (Saint Doulchard, France) were housed under conventional conditions, with food and water administered *ad libitum*, according to institutional guidelines. Twelve mice per group were immunocompromised by intraperitoneal injection of 300 mg/kg Endoxan 48 h prior to infection and then infected with a single intraperitoneal injection of 10^4^ parasites suspended in 0.3 mL of fresh IMDM medium. Where appropriate, 1 mg/mL doxycycline and 50 g/L saccharose were added every 48 h to the drinking water starting three days prior to infection. Four experimental groups were studied: animals infected with wild-type parasites without (group 1) or with doxycycline (group 2) in the drinking water, animals infected with the c *^RNAi^*PDH.ni cell line (group 3) and animals infected with the *^RNAi^*PDH.i cell line cultured for 48 h in the presence of doxycycline to pre-induce down-regulation of PDH-E2 expression and then kept with doxycycline in the drinking water (group 4). To prevent the phenotypic reversion commonly observed in BSF mutants, the injected *^RNAi^*PDH.i cell line was selected from a fresh transfection and maintained *in vitro* up to 4 weeks post-transfection before injecting the animals. Efficient down-regulation of PDH-E2 expression was confirmed by western blot and the threonine-dependency of the selected cell line was confirmed *in vitro*. The health status of the animals was monitored on a daily basis and parasitaemias were counted daily.

### Ethics statement

Experiments, maintenance and care of mice complied with guidelines of the European Convention for the Protection of Vertebrate Animals used for Experimental and other Scientific Purposes (CETS n°123). Experiments were approved by the Department for the protection of animals and plants of the Préfecture de la Gironde (Identification number A33-063-324).

## Results

### Acetate is essential for lipid biosynthesis in BSF

**Figure 2:**
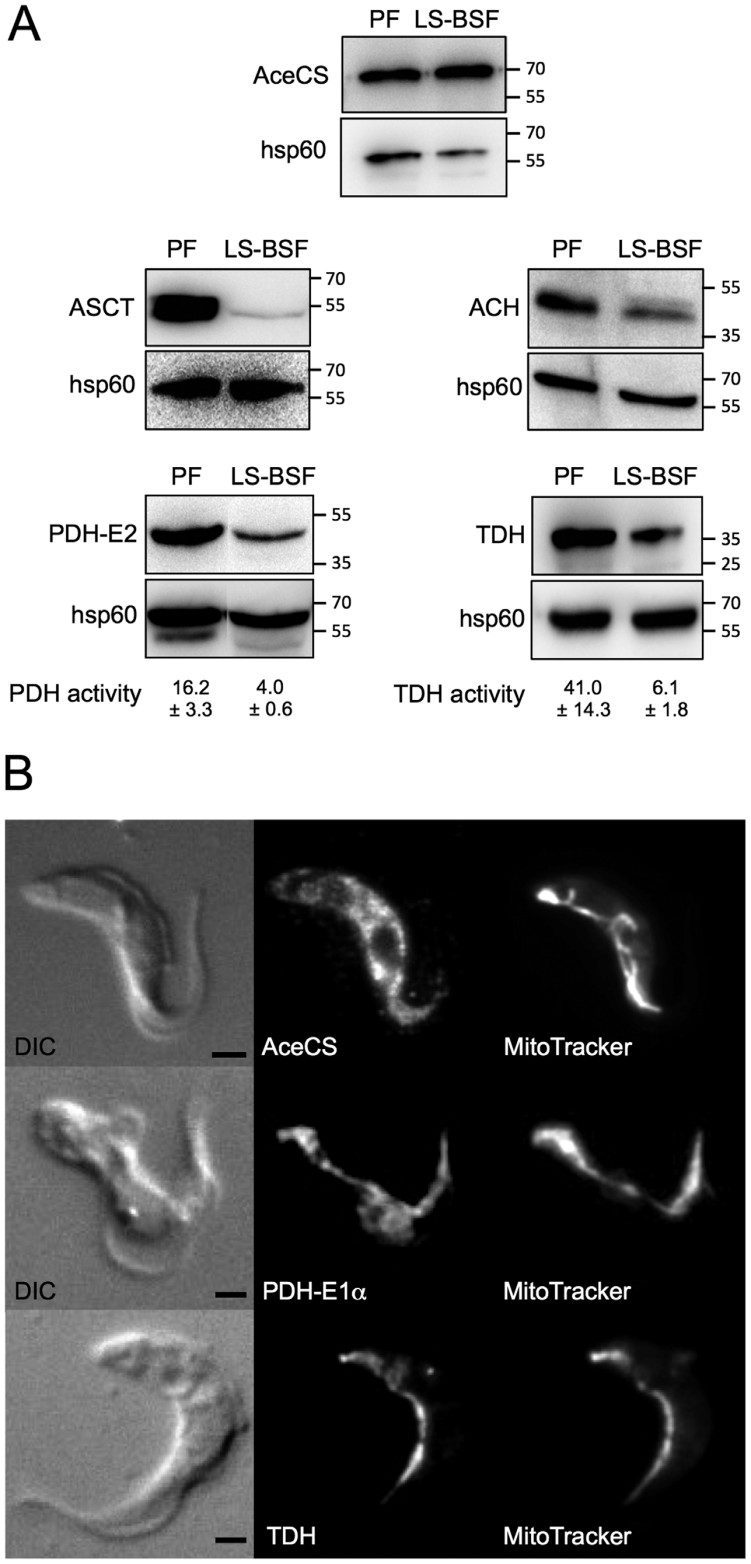
Expression and immunolocalization of enzymes involved in acetate metabolism. In Panel A, expression of AceCS, ASCT, ACH, PDH-E2 and TDH was analyzed by western blotting with specific immune sera on total protein lysates (5×10^6^ cells per lane) of wild-type 427 monomorphic bloodstream forms (LS-BSF) and procyclic cells (PF). The anti-hps60 antibodies were used as loading controls. The positions of the molecular weight markers are indicated in kDa on the right margin of each panel. The PDH and TDH activities (milliunits/mg of protein) were determined in both forms of the parasite. The “±” signs indicate SD of at least 3 independent experiments. In panel B, bloodstream cells were stained with mouse anti-AceCS (Alexa 488 channel), mouse anti-PDH-E1α (Alexa 488 channel) or rabbit anti-TDH (Alexa 488 channel) with MitoTracker as mitochondrial control. Differential interference contrast (DIC) of cells is shown to the left of each panel. Scale bar, 1 µm.

**Figure 3:**
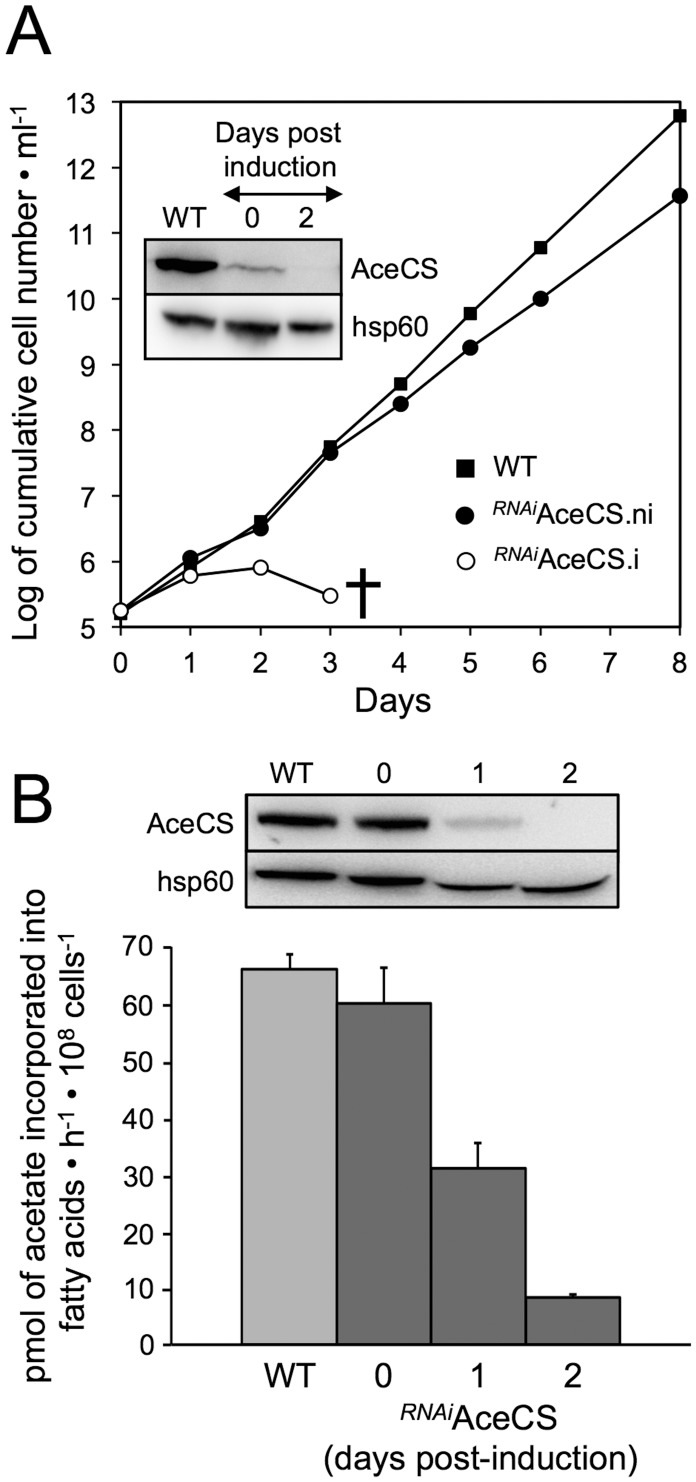
AceCS is essential for growth and lipid biosynthesis. Panel A shows growth curves of the parental 427 90-13 strain (WT) and the *^RNAi^*AceCS mutant cell line incubated in the presence (.i) or in the absence (.ni) of 10 µg/mL tetracycline. Cells were maintained in the exponential growth phase (between 2×10^5^ and 2×10^6^ cells/mL) and cumulative cell numbers reflect normalization for dilution during cultivation. The cross indicates that all cells were dead. It is to be noted that addition of 10 µg/mL tetracycline did not affect growth of the parental cell line (see Fig. 6A). Panel B shows [1-^14^C]-acetate incorporation into lipids of WT and tetracycline-induced (one and two days) and uninduced *^RNAi^*AceCS cells. ^14^C-labeled fatty acid methyl esters were separated by HPTLC after transesterification and analyzed as described in the Experimental Procedures section. The values were normalized with the amounts of total esters measured in each sample. Error bars indicate mean ± SD of 3 independent experiments.

PF depend on acetate produced in the mitochondrion to feed fatty acid biosynthesis through the essential enzyme AceCS [13]. A western blot analysis showed that AceCS (74 kDa) was expressed at the same level in the BSF and PF, and an immunofluorescence analysis using the anti-AceCS immune serum showed a homogeneous diffuse pattern characteristic of a cytoplasmic localization (Fig. 2), suggesting that this pathway may also exist in BSF. AceCS is essential for BSF viability, as demonstrated by the death of the *^RNAi^*AceCS.i cell line three days post-induction of down-regulation of the *AceCS* gene expression (.ni and .i stands for uninduced and tetracycline-induced, respectively) (Fig. 3A). To investigate the role of AceCS, radiolabel incorporation into fatty acids from [1-^14^C]-acetate was measured for the parental and *^RNAi^*AceCS cell lines incubated in the IMDM medium. Label incorporation into fatty acids was reduced 2.1- and 8.1-fold one and two days after tetracycline addition, respectively, which correlates with the reduction of AceCS expression (Fig. 3B). Altogether these data demonstrate that proliferative BSF require acetate to feed the essential fatty acid biosynthetic pathway, as previously observed in PF [13].

### BSF express enzymes involved in acetate production from glucose and threonine

The IMDM medium does not contain acetate, except the minor contribution of the 10% FCS supplement (∼5 µM) [34]. Consequently, LS-BSF may produce acetate from the catabolic pathways previously identified in PF, *i.e.* mitochondrial production of acetyl-CoA from glucose and threonine degradation through PDH and TDH, respectively [3], followed by conversion of acetyl-CoA into acetate by two mitochondrial enzymes, ASCT [9] and ACH [10]. A western blot analysis showed that ASCT (54 kDa), ACH (40.5 kDa), PDH-E2 (E2 subunit of PDH, 49.6 kDa) and TDH (39.5 kDa) are expressed in the 427 BSF strain, which has lost the ability to differentiate into SS-BSF (Fig. 2A). This was confirmed by determination of the PDH and TDH activities in LS-BSF, which were 4-fold and 6.7-fold lower than PF, respectively (Fig. 2A). Immunofluorescence analyses revealed colocalization of PDH-E1α (E1α subunit of PDH) and TDH with the mitochondrion-specific dye MitoTracker Red CMXRos (Invitrogen) (Fig. 2B). The mitochondrial localization of TDH is consistent with a 24-amino-acid N-terminal mitochondrial targeting signal predicted by MitoProt (http://ihg.gsf.de/ihg/mitoprot.html) with a high probability (0.82). Since BSF express the whole set of enzymes required for acetate production, we then used a combination of reverse genetics on PDH-E2 and TDH and metabolic profiling by NMR to investigate mitochondrial acetate production in BSF.

### BSF and PF produce equivalent amounts of acetate from glucose

**Figure 4:**
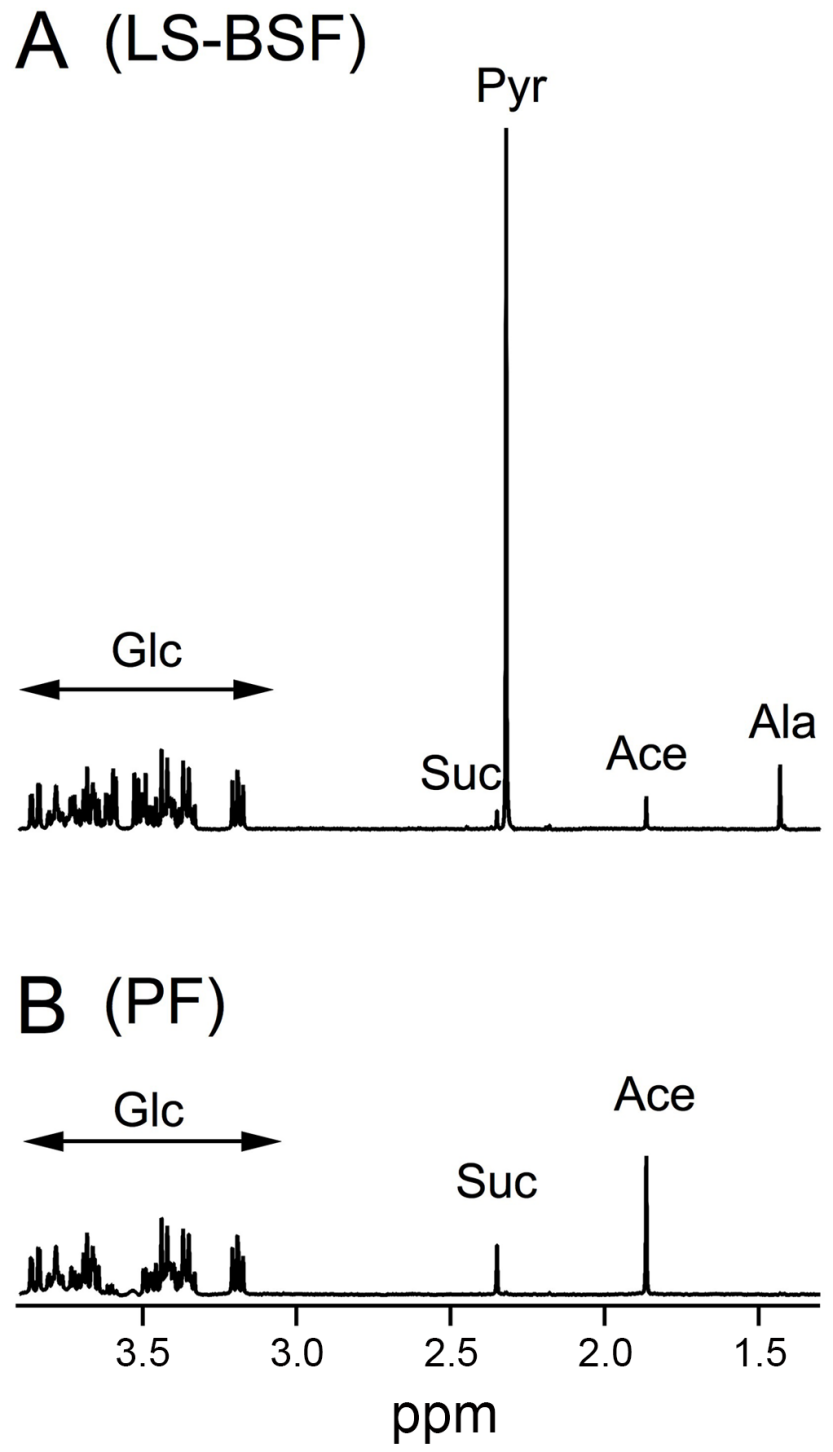
^1^H-NMR analysis of excreted end-products from glucose by LS-BSF and PF. Metabolic end-products (pyruvate, Pyr; acetate, Ace; alanine, Ala and succinate, Suc) excreted by the wild-type 427 monomorphic BSF (A) and PF cells (B) from 4 mM glucose (Glc) were determined by ^1^H-NMR. The experiment was performed in 2.5 mL of PBS with 2.5×10^7^ LS-BSF cells (A) and 5×10^7^ PF cells (B). Each spectrum corresponds to one representative experiment from a set of at least 3. A part of each spectrum ranging from 1.1 ppm to 4 ppm is shown.

**Figure 5:**
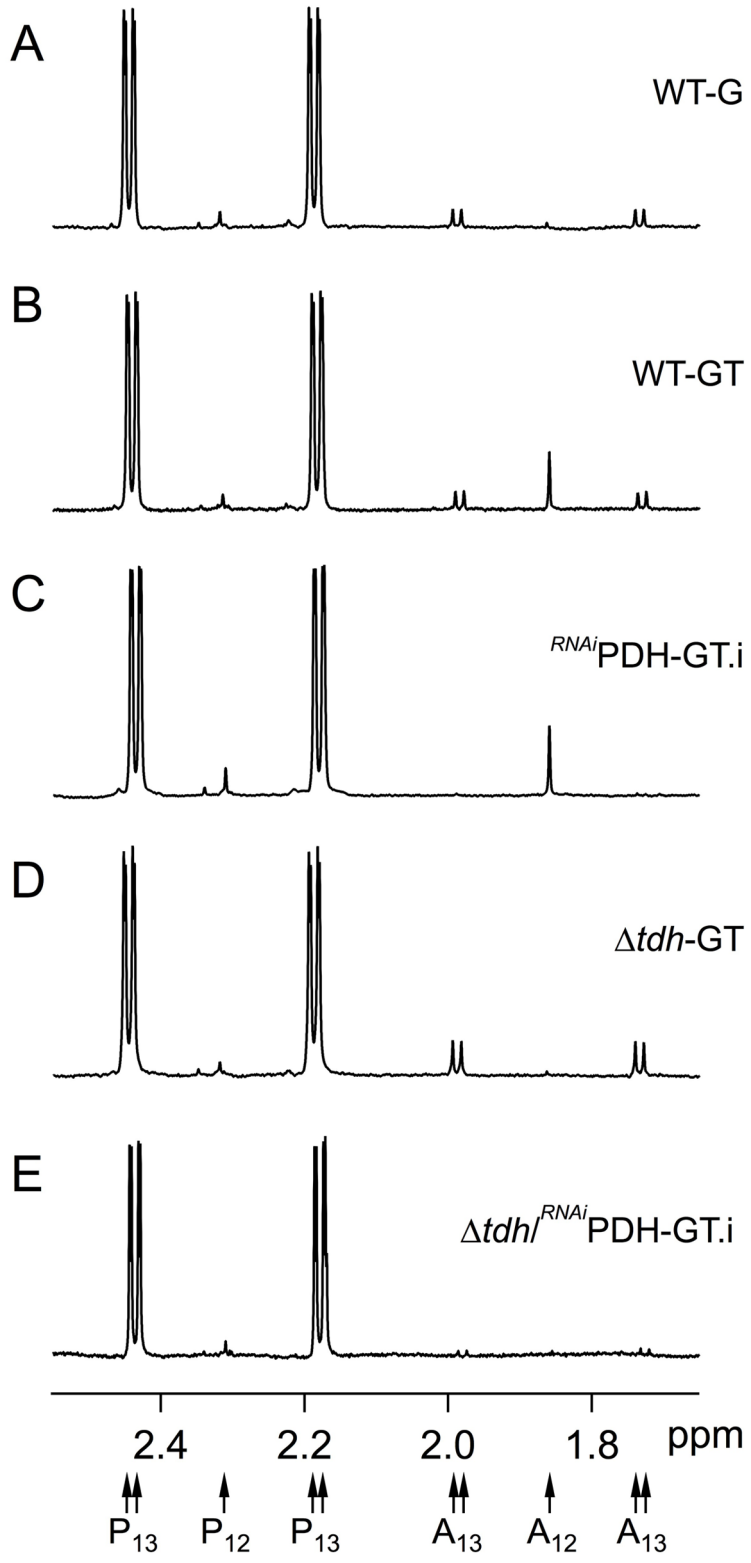
^1^H-NMR analysis of excreted end-products from glucose and threonine metabolism. Metabolic end-products (pyruvate and acetate) excreted by the wild-type BSF cell line (A–B), the tetracycline-induced *^RNAi^*PDH.i mutant (C), the Δ*tdh* mutant (D) and the tetracycline-induced Δ*tdh*/*^RNAi^*PDH.i mutant (E) from D-[U-^13^C]-glucose and/or threonine was determined by ^1^H-NMR. The cells were incubated in PBS containing 4 mM D-[U-^13^C]-glucose with (_GT) or without (_G) 4 mM threonine. Each spectrum corresponds to one representative experiment from a set of at least 3. A part of each spectrum ranging from 1.6 ppm to 2.6 ppm is shown. The resonances were assigned as indicated below the spectra: A_12_, acetate; A_13_, ^13^C-enriched acetate; P_12_, pyruvate; P_13_, ^13^C-enriched pyruvate.

It is widely considered that LS-BSF excrete only pyruvate from glucose metabolism, while PF mainly produce acetate and succinate. To compare glucose metabolism in these two forms, 2.5×10^7^ LS-BSF and 5×10^7^ PF (equivalent to 0.25 mg of proteins) were incubated in 2.5 mL of PBS containing 4 mM glucose (Fig. 4) or [U-^13^C]-glucose (Fig. 5A and Table 1). ^13^C-enriched end-products excreted in the medium from [U-^13^C]-glucose metabolism were quantified by ^1^H-NMR (Table 1). It is to note that quantification errors are significant, in particular for molecules representing less than 5% of all excreted end-products. As expected, BSF mainly converted glucose into pyruvate (7761 nmol/h/10^8^ cells), which accounts for 85.1% of the excreted end-products. In addition, BSF excreted significant amounts of alanine, acetate and succinate, which represent 9.2%, 4.9% and 0.8% of the excreted end-products from glucose metabolism, respectively (Table 1). Surprisingly, the rate of excretion of ^13^C-enriched acetate and succinate from [U-^13^C]-glucose was only 2-fold lower in BSF than in PF (446 *versus* 789 nmol of acetate/h/10^8^ cells and 71 *versus* 156 nmol of succinate/h/10^8^ cells, respectively) (Table 1, see Fig. 4). The unexpected similar rate of acetate and succinate excretion in both trypanosome forms is probably due to the ∼10-fold higher glycolytic rate in BSF (the rate of glycolytic end-product excretion was 9.4-fold higher in BSF compared to PF - Table 1). Consequently, the high glycolytic rate in BSF combined with the dominant conversion of glucose into pyruvate (85.1% of the excreted end-products) may have led to underestimation of the role of acetate and succinate production in LS-BSF, although their rate of production were in the same range in PF.

**Table 1:** Excreted end-products of glucose by *T. brucei* long-slender bloodstream form (BSF) and procyclic form (PF) cell lines.

Cell line^a^^, ^ b	n^c^	Excreted molecules from *[^13^C-U]glucose* d														
		Acetate			Succinate			Pyruvate			Alanine			TOTAL		
		*nmol/h/10^8^ cells (% of excreted molecules)*														
EATRO1125.T7T (PF)	5	789	±	170 (81.5)	156	±	43 (16.2)	23	±	44 (2.3)	ND^e^			968	±	180 (100)
427 90-13 (BSF)	9	446	±	159 (4.9)	71	±	62 (0.8)	7761	±	1878 (85.1)	837	±	230 (9.2)	9115	±	1828 (100)
*^RNAi^*PDH.ni	7	755	±	320 (7.3)	114	±	155 (1.1)	8631	±	1398 (83.1)	890	±	264 (8.5)	10390	±	1426 (100)
*^RNAi^*PDH.i	6	55	±	88 (0.7)	115	±	104 (1.4)	7288	±	2193 (89.1)	721	±	145 (8.8)	8179	±	2243 (100)
Δ*tdh*	3	510	±	100 (6.5)	33	±	11 (0.4)	6625	±	652 (84.3)	695	±	190 (8.8)	7863	±	913 (100)
Δ*tdh/^RNAi^*PDH.ni	3	300	±	34 (3.5)	12	±	12 (0.1)	7445	±	223 (86.5)	852	±	113 (9.9)	8610	±	239 (100)
Δ*tdh/^RNAi^*PDH.i	3	33	±	58 (0.5)	2	±	4 (0.02)	6350	±	460 (90.3)	649	±	48 (9.2)	7034	±	365 (100)

The extracellular PBS medium of trypanosomes incubated with 4 mM [U-^13^C]-glucose was analyzed by ^1^H-NMR spectrometry to detect and quantify excreted end-products.

a .i: RNAi cell lines tetracycline-induced for 5 to 10 days depending on the cell line and the experiments; .ni: uninduced RNAi cell lines.

b PF and BSF were incubated at the 2×10^7^ cells/mL (0.1 mg of proteins/mL) and 10^7^ cells/mL (0.1 mg of proteins/mL), respectively.

c Number of experiments.

d Mean ± SD of 3 or 9 experiments of cells incubated in PBS containing 4 mM [U-^13^C]-glucose with or without 4 mM threonine (nmol/h/10^8^ cells), and the percent of each excreted molecules (values into brackets) are presented.

e Not detectable.

**Figure 6:**
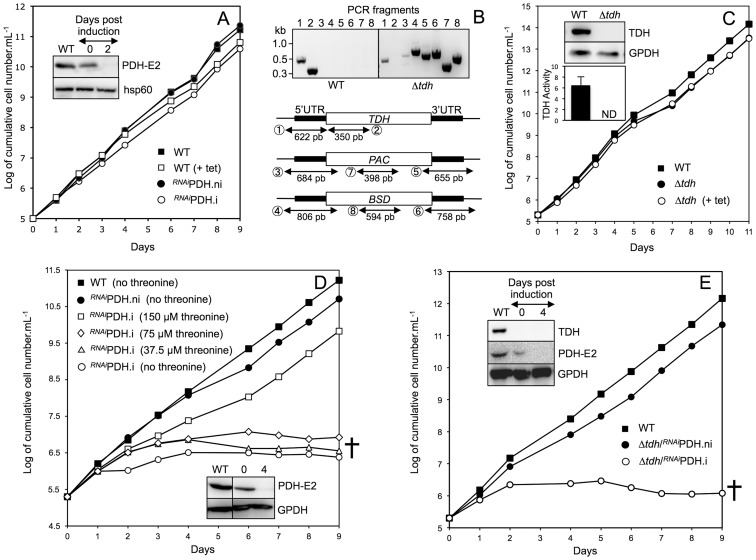
Analysis of mutant cell lines. Panels A and C–E show growth curve of the parental 427 90-13 BSF cells (WT) and mutant cell lines incubated in the IMDM medium. The mutant cell lines are *^RNAi^*PDH (A and D), Δ*tdh* (C) and Δ*tdh*/*^RNAi^*PDH (E) incubated in the presence (.i, +tet) or in the absence (.ni) of tetracycline. In panel D, the *^RNAi^*PDH.i cell line was incubated in threonine-depleted medium supplemented with 0 to 150 µM threonine. The cells were maintained in the exponential growth phase (between 2×10^5^ and 2×10^6^ cells/mL) and cumulative cell numbers reflect normalization for dilution during cultivation. The insets in panels A and C–E show western blot analyses of the parental (WT) and mutant cell lines with the immune sera indicated in the right margin. The lower inset in panel C shows the TDH activity (milliunits/mg of protein) measured in the WT and Δ*tdh* cell lines (ND stands for not detectable). Panel B shows a PCR analysis of genomic DNA isolated from the parental WT and Δ*tdh* cell lines. Amplifications were performed with primers based on sequences that flank the 5′UTR and 3′UTR fragments used to target the *TDH* gene depletion (black boxes) and internal sequences of the puromycin (*PAC*, PCR products 3 and 5) or blasticidin (*BSD*, PCR products 4 and 6) resistance genes. As controls, we also used primers corresponding to the 5′UTR (PCR product 1), the *TDH*, *PAC* and *BLE* genes (PCR products 2, 7 and 8, respectively). As expected, PCR amplification of the 5′UTR was observed for both cell lines (lane 1), while the *TDH* gene was PCR-amplified only in the WT cells (lane 2) and PCR products with marker-derived primers were observed only in the Δ*tdh* cell line (lanes 3–8).

To confirm acetate production from glucose metabolism by LS-BSF, we conducted RNAi-mediated down-regulation of expression of the *PDH-E2* gene. The *^RNAi^*PDH.i cell line showed no growth phenotype upon tetracycline induction (Fig. 6A), although the PDH-E2 protein was no longer detectable by western blot two days post-induction (Fig. 6A, inset). Metabolite profiling of the *^RNAi^*PDH.i cell line incubated in the presence of 4 mM of [U-^13^C]-glucose showed a 13.7-fold reduction of acetate production from glucose compared to the *^RNAi^*PDH.ni cells (55 *versus* 755 nmol/h/10^8^ cells) (Table 1). It is to note that, for unknown reasons, the uninduced *^RNAi^*PDH.ni cell line produces ∼1.7-times more acetate from glucose than the parental cells.

### Mitochondrial production of acetate is essential for BSF

Since both BSF and PF have been reported to produce acetate from threonine [12], which is present in the IMDM medium (0.9 mM), we investigated the threonine degradation pathway in BSF. Incubation of the parasites in threonine-depleted medium, which contains only ∼15 µM of the amino acid coming from FCS [35], did not affect growth of the wild-type and *^RNAi^*PDH.ni cells, while growth of the *^RNAi^*PDH.i mutant was abolished (Fig. 6D). To confirm that glucose and threonine degradations contribute to acetate production, both pathways were interrupted by down-regulating PDH-E2 expression in the TDH null background (Δ*tdh*/*^RNAi^*PDH cell line). First, both *TDH* alleles were replaced by the puromycin (*PAC*) and blasticidin (*BSD*) markers in the Δ*tdh* cell line, with no effect on growth rate (Fig. 6C). Deletion of both *TDH* alleles was confirmed by PCR analyses (Fig. 6B), western blot analyses and enzymatic assays (insets of Fig. 6C). Second, RNAi-mediated down-regulation of PDH-E2 was performed in the Δ*tdh* background. Growth of the Δ*tdh*/*^RNAi^*PDH.i cell lines was abolished three days post-induction before cell death seven days later (Fig. 6E). Addition of 4 mM acetate in the medium does not rescue growth of the Δ*tdh*/*^RNAi^*PDH.i mutant (data not shown) suggesting that acetate and/or acetyl-coA need to be produced inside the mitochondrion to feed the essential mitochondrial fatty acid pathway, may be through the production of the precursor butyryl-CoA [36]. This result confirms that abolition of mitochondrial acetyl-CoA/acetate production from both glucose and threonine is lethal for BSF grown in standard medium.

### Relative contribution of glucose and threonine to acetate production

To address this question we developed a metabolite profiling assay based on the ability of ^1^H-NMR spectrometry to distinguish ^13^C-enriched molecules from ^12^C ones. Cells were incubated in PBS with equal amounts (4 mM) of [U-^13^C]-glucose and unenriched threonine in order to perform a quantitative analysis of threonine-derived and glucose-derived acetate production by ^1^H-NMR. When [U-^13^C]-glucose was the only carbon source in the incubation medium, the excreted [^13^C]-acetate (annotated A_13_ in Fig. 5) was represented by two doublets with chemical shifts at around 2.0 ppm and 1.75 ppm, respectively (see Fig. 5A). It is to be noted that threonine metabolism cannot be analyzed independently since glucose is essential for BSF. Addition of threonine to the [U-^13^C]-glucose/PBS medium induced production of threonine-derived [^12^C]-acetate (386 nmol/h/10^8^ cells) in addition to [^13^C]-glucose-derived [^13^C]-acetate (532 nmol/h/10^8^ cells) (Fig. 5B and Table 2). This shows that in the presence of equal amounts of both carbon sources, glucose contributes ∼1.4-fold more than threonine to acetate production.

**Table 2:** Excreted end-products of glucose and threonine metabolism by *T. brucei* long-slender bloodstream form (BSF) and procyclic form (PF) cell lines.

Cell line^a^^, ^ b	n^c^	Excreted acetate from threonine^d^			Excreted molecules from *[^13^C-U]Glucose* d ^,^ e															Ratio acetate (Glu)/(Thr)^f^
					Acetate			Succinate			Pyruvate			Alanine			TOTAL			
		*nmol/h/10^8^ cells (% of excreted molecules)*																		
EATRO1125.T7T (PF)	5	1306	±	210	668	±	127 (55.2)	308	±	92 (25.5)	207	±	98 (17.1)	27	±	60 (1.1)	1210	±	145 (100)	0.51
427 90-13 (BSF)	9	386	±	59	532	±	111 (3.8)	76	±	68 (0.6)	11964	±	2161 (86.0)	1342	±	266 (9.6)	14070	±	2437 (100)	1.38
*^RNAi^*PDH.ni	6	423	±	125	847	±	412 (6.5)	102	±	64 (0.8)	10918	±	3177 (83.4)	1223	±	536 (9.3)	13090	±	3496 (100)	2.00
*^RNAi^*PDH.i	5	373	±	211	16	±	62 (0.1)	189	±	166 (1.6)	10723	±	3344 (88.5)	1191	±	530 (9.8)	12119	±	4185 (100)	0.04
Δ*tdh*	3	ND^g^			899	±	169 (7.5)	39	±	19 (0.3)	9879	±	186 (81.7)	1272	±	451 (10.5)	12090	±	525 (100)	>100
Δ*tdh/^RNAi^*PDH.ni	3	ND			488	±	72 (4.3)	12	±	5 (0.1)	9595	±	348 (84.5)	1266	±	82 (11.1)	11361	±	479 (100)	>100
Δ*tdh/^RNAi^*PDH.i	3	ND			109	±	61 (1.2)	ND			8098	±	744 (88.3)	965	±	206 (10.5)	9172	±	946 (100)	>100

The extracellular PBS medium of trypanosomes incubated with 4 mM [U-^13^C]-glucose and 4 mM threonine was analyzed by ^1^H-NMR spectrometry to detect and quantify excreted end-products.

a .i: RNAi cell lines tetracycline-induced for 5 to 10 days depending on the cell line and the experiments; .ni: uninduced RNAi cell lines.

b PF and BSF were incubated at the 2×10^7^ cells/mL (0.1 mg of proteins/mL) and 10^7^ cells/mL (0.1 mg of proteins/mL), respectively.

c Number of experiments.

d Mean ± SD of 3 or 9 experiments of cells incubated in PBS containing 4 mM [U-^13^C]-glucose and 4 mM threonine (nmol/h/10^8^ cells) are presented.

e Values into brackets represent percent of each excreted molecules from [U-^13^C]-glucose metabolism.

f Ratio between glucose-derived and threonine-derived [^13^C]-acetate excreted.

g Not detectable.

^1^H-NMR metabolite profiling of the single and double mutants confirmed the involvement of both glucose and threonine in acetate production. As expected, production of [^13^C]-glucose-derived [^13^C]-acetate was ∼50-times lower in the *^RNAi^*PDH.i than in the *^RNAi^*PDH.ni cells (16 *versus* 847 nmol/h/10^8^ cells), while threonine-derived acetate production was not affected. Conversely, production of threonine-derived acetate was abolished in the Δ*tdh* mutant, while [^13^C]-glucose-derived [^13^C]-acetate was not affected (Table 2 and Fig. 5C–D). Finally, production of acetate from both carbon sources was affected in the Δ*tdh*/*^RNAi^*PDH.i double mutant cell line (Table 2 and Fig. 5E).

### Threonine contributes to acetate production in BSF *in vivo*

BALB/c mice immunocompromised by Endoxan treatment were injected with wild-type and *^RNAi^*PDH cells and kept with or without doxycycline, a stable tetracycline analog, in the drinking water to down-regulate expression of PDH-E2. Animal survival and the blood parasite levels were monitored. No differences were observed between the four groups of animals, in which parasite density started to rise at day three post-infection. All mice were dead at days 6–7 post-infection (data not shown). This shows that acetate production from glucose is not necessary for the viability of *T. brucei in vivo*, suggesting that a possible acetate source (threonine) that is present in the blood is absent in the threonine-depleted *in vitro* culture medium. As mentioned above, mammalian blood contains approximately 150 µM threonine [35], [37], which is 10-times higher than in the threonine-depleted IMDM medium. The *^RNAi^*PDH.i cell line died in IMDM medium containing 15, 37.5 and 75 µM threonine, while addition of 150 µM of the amino acid restored its growth *in vitro* (Fig. 6D), suggesting that the homeostatic threonine blood concentration (150 µM) is sufficient to provide BSF with the required acetyl-CoA/acetate molecules. Altogether, this demonstrates that BSF trypanosomes have developed two complementary and self-sufficient ways to maintain the essential production of acetate in the blood of mammalian hosts.

## Discussion

LS-BSF trypanosomes are well known for their glucose-dependency to satisfy ATP requirements [38]. Indeed, net production of all cellular ATP is fulfilled by the last glycolytic step catalyzed by pyruvate kinase, which produces pyruvate, the excreted glycolytic end-product. Excretion of significant amounts of other partially oxidized end-products of glycolysis, such as glycerol, succinate and alanine, has been previously reported [14], [17], [39], [40]. However, in the late seventies emerged a general dogma whereby pyruvate was considered the exclusive glycolytic end-product excreted from LS-BSF under aerobic conditions [15], [16], because the minor end-products were assigned to either non-growing conditions or contamination with non-dividing SS-BSF [18], [19]. Here, we used as an experimental model the 427 BSF strain, which has lost the ability to differentiate into SS-BSF, in order to focus our analysis of glucose metabolism on LS-BSF trypanosomes. This laboratory-adapted monomorphic strain is insensitive to the stumpy inductor factor, but, it successfully differentiates *in vitro* into *bona fide* SS-BSF, for instance when expression of the protein kinase target of rapamycin (TOR4) is inhibited [41]. This suggests that the 427 strain can be considered as a slender-like BSF that has lost the ability to respond to the stumpy inductor factor, and as such is the relevant model to study the metabolism of proliferative BSF. Our metabolic analyses showed that LS-BSF can produce almost as much succinate and acetate from glucose as PF incubated in the same conditions. This suggests that most, if not all, enzymes involved in the “succinate and acetate branches” previously characterized in PF are also expressed in LS-BSF. To produce acetate, PDH (step 1 in Fig. 1) converts pyruvate into acetyl-CoA, which is the substrate of ASCT (step 2) and ACH (step 3) for acetate production. ASCT expression is low in BSF (Fig. 2 and [19]), while ACH is relatively abundant (Fig. 2) with an ACH activity ∼2-fold higher than PF (data not shown). Three of the four PDH subunits have been investigated so far and are expressed in BSF (PDH-E1α and PDH-E2, see Fig. 2; PDH-E3, [42]), with a PDH enzymatic activity only 4-fold lower than in PF (Fig. 2). This relatively high PDH activity is in agreement with a recent comparative SILAC proteomics analysis showing that PDH-E1α, PDH-E1ß, PDH-E2 and PDH-E3 are 5.3-, 7.6-, 5.2- and 8.1-fold more abundant in PF than LS-BSF, respectively [43]. The same proteomics analysis in LS-BSF also detected most, if not all, of the enzymes involved in succinate production from phosphoenolpyruvate, although at a lower level of expression than in PF (between 3- and 20-fold). Altogether, this clearly demonstrates that LS-BSF have maintained the capacity to produce and excrete acetate and succinate from glycolysis. The relatively high rate of acetate and succinate production (only ∼2-fold higher in PF), while the enzymes involved in the corresponding metabolic pathways are 5- to 20-times more abundant in PF, may be due to the considerably higher glycolytic flux in BSF. We determined that the excretion rate of glycolytic end-products is 9.5-fold higher in BSF than in PF (9115 *versus* 968 nmol of excreted end-products/h/10^8^ cells), which is consistent with the previously measured 5- to 10-fold difference in glycolytic flux [14]. A recent analysis of the glycolytic flux in the same BSF strain incubated in growing conditions (IMDM containing 20 mM glucose) showed a higher rate of pyruvate production compared to our analysis performed in PBS containing 4 mM glucose and threonine (19.2 *versus* 12.0 µmol/h/10^8^ cells) [44]. The reduced glycolytic flux observed in PBS conditions certainly reflects the difference between non-growing conditions (PBS) and exponential growth (IMDM) with a doubling time in the range of 5 h [44]. Also, the 35% reduction in total end-product fluxes for the wild type cells depending on the available substrates (PBS/glucose *versus* PBS/glucose/threonine) shows that metabolic fluxes are dependent on the exact context of substrates present (Table 1).

It is important to note that our experimental procedures do not reflect physiological conditions, since trypanosomes were incubated at high density in PBS containing 4 mM glucose. Consequently, these minor glycolytic end-products might be excreted at a lower rate, or not at all, by LS-BSF trypanosomes *in vivo*. A recent quantitative analysis of the fate of glucose in exponentially growing 427 LS-BSF *in vitro* (the same strain analysis here) showed that pyruvate is the only excreted glycolytic end-product [44]. Glucose, pyruvate and glycerol were analysed in that study. Although they report an almost complete carbon balance between glucose uptake and pyruvate excretion, their analysis leaves room for small fluxes towards products they did not analyse such as acetate and succinate. We here report these end-products, with fluxes to acetate and succinate together representing ∼5% of the excreted glycolytic end products. The exact fraction of total carbon going to these end-products is difficult to assign, due to the errors in quantification of fluxes and because our results did not enable the calculation of a carbon balance between carbon uptake and excretion. Whatever the rate of acetate excretion from glucose metabolism in exponentially growing LS-BSF is, its production is essential for growth, as exemplified by the death of the *^RNAi^*PDH.i cell line incubated in the absence of threonine, the other acetate source. This was confirmed by inducing cell death upon blocking acetate production from both carbon sources in the Δ*tdh*/*^RNAi^*PDH.i double mutant, while growth of the corresponding single mutants in standard IMDM medium was not affected (Fig. 6). The relevance of acetate production from glycolysis for LS-BSF is further strengthened by (*i*) the same high rate of growth of the wild-type parasite, even in the absence of threonine (Fig. 6), as recently observed by the development of a new minimal medium that supports growth of BSF [37] and (*ii*) the impossibility to rescue growth of the Δ*tdh*/*^RNAi^*PDH.i double mutant by addition of sodium acetate in the medium, which cannot substitutes glucose-derived acetate production.

The above-mentioned reverse genetic experiments combined with ^1^H-NMR metabolic analyses also clearly demonstrate that glucose and threonine are the only significant carbon sources contributing to the essential production of acetate in the 427 BSF strain. To our knowledge, this is the first report showing acetate production from glucose in LS-BSF, while threonine has been described before as an acetate-source in BSF [12], [45]. Inhibition of a single acetate-production pathway in the *^RNAi^*PDH.i and Δ*tdh* cell lines grown in standard IMDM medium does not affect growth of LS-BSF, indicating that a single active pathway is sufficient for growth *in vitro*. This is probably also true *in vivo*, since (*i*) glucose concentration is higher in the blood than in our experimental conditions (5 *versus* 4 mM) and (*ii*) the relatively low homeostatic concentration of threonine in mammalian blood (150 µM) is sufficient for acetate production, as demonstrated by the absence of growth effect of the *^RNAi^*PDH.i mutant in medium containing at least 150 µM threonine (Fig. 6D). When incubated with equal amounts of threonine and glucose (4 mM), the parasite produces ∼1.4-fold more acetate from glycolysis. Mammalian blood contains ∼30-fold more glucose than threonine (5 mM *versus* 150 µM), which strengthens the view that the contribution of glucose to acetate production is relevant *in vivo*. This contrasts with an equivalent recent analysis performed on the procyclic form of *T. brucei*, which prefers threonine for acetate and fatty acid productions, with a ∼2.5-fold higher contribution of threonine compared to glucose when incubated with 4 mM of both carbon sources [46].

In PF trypanosomes, the mitochondrial production of acetate is essential to feed *de novo* fatty acid biosynthesis through the “acetate shuttle” [13]. In this shuttle, acetate produced in the mitochondrion reaches the cytosol, where a part of it is converted by AceCS into acetyl-CoA to produce malonyl-CoA, the elongator for fatty acid biosynthesis. It is noteworthy that both the microsomal elongase-dependent and mitochondrial type II fatty acid synthase pathways use malonyl-CoA to elongate fatty acids [47]. As observed in PF, AceCS is essential for incorporation of [1-^14^C]-acetate into LS-BSF fatty acids (Fig. 3), indicating that AceCS is required for *de novo* fatty acid synthesis. In addition, Gilbert *et al.* previously showed that both glucose and threonine are used as carbon sources for fatty acid synthesis [47]. Altogether, this demonstrates that, like PF, proliferative BSF trypanosomes use the “acetate shuttle” to feed fatty acid biosynthetic pathways. The essential role of mitochondrial fatty acid synthesis has been documented in BSF, however, RNAi-mediated down regulation of elongase genes involved in the microsomal fatty acid biosynthesis does not affect growth of the parasite [36], [48], [49], [50]. Consequently, the lethal phenotype observed for the LS-BSF *^RNAi^*AceCS mutant is probably due to the dramatic reduction of cytosolic malonyl-CoA production required to feed mitochondrial fatty acid biosynthesis, which contribute to ∼10% of cellular fatty acid production [36].

As mentioned above, our detection of several “minor” glycolytic end-products excreted by BSF trypanosomes is consistent with most, if not all, early reports [14], [17], [39], [40] and the recent quantitative flux analysis in BSF 427 [44]. The high glycolytic flux combined with the almost exclusive conversion of glucose into pyruvate has probably caused the community to overlook these minor end-products and the metabolic pathways leading to their production, although their biosynthesis may be essential for the parasite, as described here for acetate. For instance, we also observed that LS-BSF excretes alanine from glucose (twice more than acetate, Table 1), as reported before [17]. Alanine is produced from pyruvate by transfer of an amino group coming from various possible amino acid sources. A recent metabolomic analysis showed that glutamate and hydrophobic keto acids accumulate in the BSF spent media, suggesting that glutamine and hydrophobic amino acids are possible substrates of alanine aminotransferase for alanine production [37]. The relevance of alanine production from glycolysis is strengthened by the reported accumulation of hydrophobic keto acids in the plasma and urine of infected rodents [51], [52] and the requirement of alanine aminotransferase activity for *in vitro* growth of the parasite [53]. Succinate production from glycolysis may also be of importance for biosynthetic pathways in LS-BSF, as exemplified by the requirement of fumarate for *de novo* synthesis of pyrimidine through the unusual fumarate-dependent dihydroorotate dehydrogenase [54]. Altogether, this highlights the need to reconsider and further investigate the metabolic pathways leading to minor glycolytic end-products, which are still considered negligible compared to the total carbon flux from glucose [44], in order to reveal new essential metabolic pathways that could be targeted to develop new trypanocidal molecules. Beyond glycolysis, other overlooked metabolic pathways of the central metabolism need to be revisited in LS-BSF, as exemplified by the recent observation that RNAi down-regulation of the tricarboxylic acid enzyme succinyl-CoA synthetase induces one of the most spectacular death phenotypes observed in BSF, with 100% cell death within less than 20 h post-induction [55], while this pathway is considered repressed in BSF.
